# Sleep Patterns in Midlife and Brain Age

**DOI:** 10.1002/alz.085643

**Published:** 2025-01-09

**Authors:** Clémence Cavaillès, Christina S. Dintica, Mohamad Habes, Yue Leng, Mercedes Carnethon, Kristine Yaffe

**Affiliations:** ^1^ Department of Psychiatry and Behavioral Sciences, University of California, San Francisco, San Francisco, CA USA; ^2^ Neuroimage Analytics Laboratory and Glenn Biggs Institute Neuroimaging Core, Glenn Biggs Institute for Neurodegenerative Diseases, University of Texas Health San Antonio, San Antonio, TX USA; ^3^ Center for AI and Data Science for Integrated Diagnostics and Center for Biomedical Image Computing and Analytics, University of Pennsylvania, Philadelphia, PA USA; ^4^ Northwestern University Feinberg School of Medicine, Chicago, IL USA; ^5^ Department of Neurology, University of California, San Francisco, San Francisco, CA USA; ^6^ Department of Epidemiology, University of California, San Francisco, San Francisco, CA USA

## Abstract

**Background:**

Previous studies have linked sleep disturbances with an increased risk of dementia among older adults. However, the association between sleep patterns and brain health earlier in life is less understood. We aimed to determine how sleep in early midlife relates to an MRI‐derived indicator of brain age in late midlife.

**Method:**

We analyzed 619 participants from the Coronary Artery Risk Development in Young Adults (CARDIA) study. At the study baseline (2000‐2001), participants answered six binary questions about their typical sleep patterns, including general sleep quality, sleep duration, daytime sleepiness, trouble falling asleep, waking up at night, and early awakening. We quantified the number of poor sleep characteristics (range from 0 to 6) and categorized them into three groups (0‐1; 2‐3; >3). On average 15 years later (2015‐2016), brain MRIs were obtained, and a high dimensional pattern analysis was used to determine brain age by quantifying individual differences in age‐related atrophy. We used multivariable linear regressions to examine the association between sleep patterns and brain age.

**Result:**

At baseline, the participants’ mean age was 40.4 (±3.4) years, 52% were women, and 38% were Black. Around 70% of participants reported 0‐1, 22% reported 2‐3, and 8% reported >3 poor sleep characteristics. After adjusting for demographics, lifestyle factors, and comorbidities, participants reporting 2‐3 and >3 poor sleep characteristics had 1.9‐year (95% confidence interval (CI) = 0.54;3.16) and 3.1‐year (95%CI = 1.14;5.11) greater brain age, respectively, compared with participants reporting 0‐1 poor sleep characteristic. Of the individual questions, sleep quality, trouble falling asleep, early awakening, and waking up at night were associated with a 1.7 (95%CI = 0.25;3.16), 1.6 (95%CI = 0.06;3.08), 2.1 (95%CI = 0.71;3.50), and 1.2 (95%CI = 0.08;2.23) years older brain age, respectively.

**Conclusion:**

These findings suggest an association of poor sleep in early midlife with advanced brain age 15 years later. Further research is needed to clarify the underlying mechanisms linking sleep to brain aging earlier in life.

